# Pulmonary artery aneurysm and tuberous sclerosis: case report

**DOI:** 10.31744/einstein_journal/2026RC0847

**Published:** 2026-01-28

**Authors:** Heli Vieira Brandão, Carlos Inácio Carneiro Dias, Millena Vieira Brandão Moura

**Affiliations:** 1 Universidade Estadual de Feira de Santana Feira de Santana BA Brazil Universidade Estadual de Feira de Santana, Feira de Santana, BA, Brazil.; 2 Hospital Inácia Pinto dos Santos Feira de Santana BA Brazil Hospital Inácia Pinto dos Santos, Feira de Santana, BA, Brazil.; 3 Universidade de São Paulo São Paulo SP Brazil Universidade de São Paulo, São Paulo, SP, Brazil.

**Keywords:** Tuberous sclerosis, Pulmonary artery, Aneurysm, Child

## Abstract

Tuberous sclerosis is a rare and serious genetic disease, classically characterized by epilepsy, mental deficiency, and sebaceous adenomas; however, it may also be associated with the development of multiple tumors in various organs. Pulmonary artery aneurysm has rarely been described in the literature. This article reports a case of tuberous sclerosis in a 3 year old child with a pulmonary artery trunk aneurysm, cardiac rhabdomyomas, and a bicuspid aortic valve, a combination that has been rarely described in the literature, and discusses the importance of early diagnosis, management, and treatment.

## INTRODUCTION

Tuberous sclerosis (TS) is a rare genetic degenerative disease caused by mutations in the TSC1 or TSC2 genes located on chromosomes 9 and 16, respectively. It was first described in 1880 by Desiré-Magloire Bourneville.^(1)^ Multiple tumours can affect several organs, including the brain, heart, eyes, lungs, skin, liver, and kidneys. Classic clinical manifestations include epilepsy, mental deficiency, and sebaceous adenomas; however, other symptoms may occur depending on the affected organ.^(2)^ Vascular wall abnormalities resulting from the dysplastic nature of TSC can affect the pulmonary artery, leading to aneurysm formation.^(3)^ Aneurysms of the aorta artery have been described in the literature and may occur at any age group,^(4)^ however, pulmonary artery aneurysms have rarely been described in the literature, with an incidence of 8 per 109,575 autopsies.^(5)^ Aneurysms of the pulmonary artery trunk are exceedingly rare and sparsely reported.^(6,7)^ We report the case of a child with TS presenting with a pulmonary artery aneurysm, cardiac rhabdomyoma, and a bicuspid aortic valve.

## CASE REPORT

A 3 years old male child presented with wheezing and dyspnea on exertion, as well as dyspnea during sleep, since the first year of life, with several emergency visits. He had a history of seizures one year earlier and was receiving phenobarbital for seizure control. There was no parental history of asthma. At 3 years of age, he developed a respiratory infection with worsening dyspnea and wheezing, required hospitalization, and was diagnosed with TS during follow-up at the Paediatric Hospital and paediatric cardiology service. At 7 years of age, he developed facial angiofibromas, hypomelanotic macules, and progressive dyspnea on exertion, accompanied by cold extremities.

On physical examination, the initial weight was 14 kg, RR was 32irpm, HR was 80bpm, BP was 80x40mmHg, and SatO2 was 82% in ambient air. Respiratory examination revealed intercostal retractions and stridor, more prominent over the anterior region of the left hemithorax. Cardiovascular examination showed a regular two-beat heart rhythm and a systolic murmur in the pulmonary area. He was treated with antibiotics and supplemental oxygen, with clinical improvement; however, the stridor persisted.

**Figure 1:**
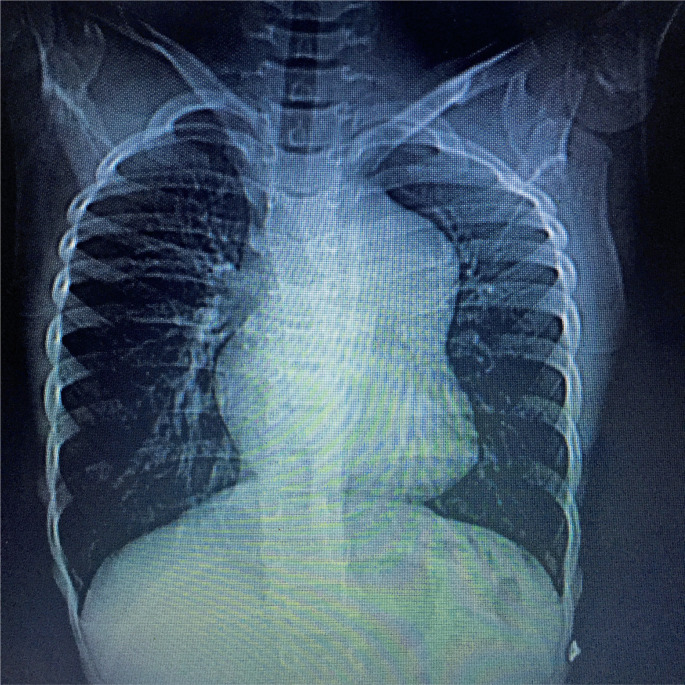
Chest x-ray

Complementary examination during hospitalization: Chest radiography revealed enlargement of the superior mediastinum with bulging of the middle arch (Figure 1).

**Figure 2:**
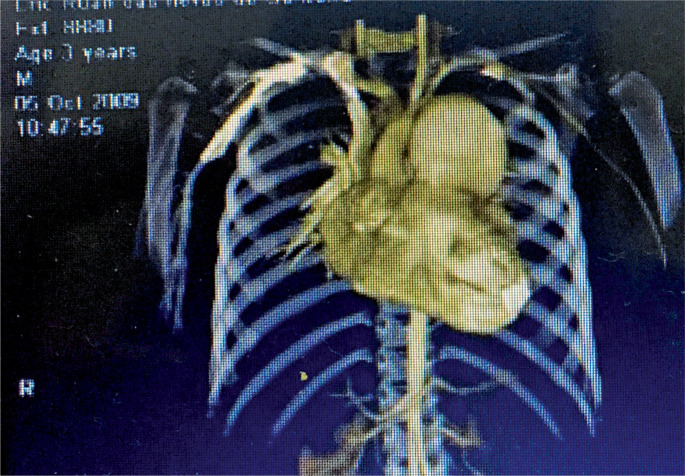
Chest CT angiography

Chest angio-tomography demonstrated mediastinal widening in pulmonary artery topography and aneurysmal dilatation of the pulmonary artery trunk along its entire length, measuring 55 x 51mm, with compression of the left main bronchus, and rhabdomyomas at the apex of the right ventricle (Figure 2). Transthoracic echocardiography showed aneurysmal dilatation of the pulmonary artery trunk, a pulmonary artery pressure of 16mmHg, multiple rhabdomyomas in the apical region of the right ventricle, one measuring 9.8mm x 15mm and two measuring 4.3mm x 6.3mm, moderate pulmonary valve insufficiency, a bicuspid aortic valve, and mild aortic valve insufficiency. Cranial magnetic resonance imaging revealed several subependymal nodules. During serial echocardiographic follow-up, progressive enlargement of the pulmonary artery trunk aneurysm was observed (56mm x 65mm), with an increase in pulmonary artery pressure to 29mmHg. At 10 years of age, surgical treatment was performed to correct the aneurysm because of the high risk of aneurysm rupture.

The article was approved by the Research Ethics Committee of the Universidade *Estadual de Feira de Santana*, CAAE: 63212722.5.0000.0053; #6.017.450.

## DISCUSSION

The characteristic triad of TS was described in 1908 by Vogt and comprises seizures, cognitive impairment, and facial angiofibromas.^(1)^ The skin and central nervous system are the most commonly affected systems involved in approximately 95% and 90% of patients, respectively.^(8)^ Cardiac manifestations are present in approximately 50-60% of patients with TS, with rhabdomyomas being the most common findings^(9)^ however, pulmonary artery aneurysms are rarely associated with TS.^(7)^ In the present case, pulmonary artery aneurysm, cardiac rhabdomyomas, and a bicuspid aortic valve were observed in association with TS.

Pulmonary artery aneurysms may be congenital or acquired and are most commonly related to vessel wall weakness due to connective tissue abnormalities such as Ehlers-Danlos syndrome or Marfan syndrome, and to left-to-right shunts that result in elevated pulmonary artery pressure, such as ventricular or atrial septal defect and ductus arteriosus. Acquired causes may be related to bacterial infections, known as Rasmussen aneurysms, and vasculitides, such as Behcet's syndrome and Hughes-Stovin syndrome.^(10)^

In TS, pulmonary artery aneurysms are most often diagnosed incidentally, and associated symptoms are due to the compression of neighboring structures caused by the dysplastic nature of the disease. In the present case, the aneurysm compressed the left main bronchus, leading to exertional dyspnea and wheezing. The diagnosis was established using chest angiotomography and echocardiography, which demonstrated a pulmonary artery aneurysm, a bicuspid aortic valve, and cardiac rhabdomyomas. Burrows and Johnson reported a case of primary pulmonary artery aneurysm and cyst in the right lung in a 52-year-old man with TS.^(6)^ Carette et al. reported a case of TS in a 57-year-old woman with a pulmonary artery aneurysm and angiolipomas in the kidneys. Histological analysis of the pulmonary artery showed proliferation of arterial musculature cells and disorganization with proliferation of elastic fibers.^(11)^ In the clinical case described here, the aneurysm was diagnosed at 3 years of age. Aneurysm treatment can be conservative or surgical,^(2,11)^ close monitoring is indicated to assess the growth of the pulmonary artery aneurysm and pulmonary artery pressure; in this case, there was an increase in the size of the aneurysm and pressure in the pulmonary artery, with a risk of rupture of the aneurysm, and a decision was made to repair the aneurysm surgically. To the best of our knowledge, this is the first reported pediatric case of TS associated with a pulmonary artery trunk aneurysm, right ventricular rhabdomyomas, and a bicuspid aortic valve.

## CONCLUSION

Pulmonary artery aneurysm in tuberous sclerosis was diagnosed early, and treatment management improved respiratory symptoms and prevented aneurysm rupture.

## Data Availability

The underlying content is contained within the manuscript and The content is already available.

## References

[1] Webb DW, Thomas RD, Osborne JP (1993). Cardiac rhabdomyomas and their association with tuberous sclerosis. Arch Dis Child.

[2] Söğüt A, Ozmen M, Sencer S, Calişkan M, Aydinli N, Ertuğrul T (2002). Clinical features of tuberous sclerosis cases. Turk J Pediatr.

[3] Paraf F, Bruneval P (1996). Dysplasie fibromusculaire arterielle et sclerosis tuberous sclerosis de Bourneville. Ann Pathol.

[4] Jost CJ, Gloviczki P, Edwards WD, Stanson AW, Joyce JW, Pairolero PC (2001). Aortic anerysms in children and young adults with tuberous sclerosis: report of two cases and review of the literature. J Vasc Surg.

[5] Izumida S, Kawano H, Tsuneto A, Doi Y, Maemura K (2020). Pulmonary Artery Aneurysm Associated with Bicuspid Pulmonary Valve. Intern Med.

[6] Dunet V, Qanadli SD, Lazor R, Beigelman-Aubry C (2013). Multiple pulmonary artery aneurysms in tuberous sclerosis complex. BMJ.

[7] Burrows NJ, Johnson SR (2004). Pulmonary artery aneurysm and tuberous sclerosis. Thorax.

[8] Rosser T, Panigrahy A, McClintock W (2006). The diverse clinical manifestations of tuberous sclerosis complex: a review. Semin Pediatr Neurol.

[9] Quek SC, Yip W, Quek ST, Chang SK, Wong ML, Low PS (1998). Cardiac manifestations in tuberous sclerosis: a 10-year review. J Paediatr Child Health.

[10] Bartter T, Irwin RS, Nash G (1988). Aneurysms of the pulmonary arteries. Chest.

[11] Carette MF, Antoine M, Bazelly B, Cadranel J, Khalil A (2006). Primary pulmonary artery aneurysm in tuberous sclerosis: CT, angiography and pathological study. Eur Radiol.

